# Cytokine profile of autologous conditioned serum for treatment of osteoarthritis, *in vitro *effects on cartilage metabolism and intra-articular levels after injection

**DOI:** 10.1186/ar3050

**Published:** 2010-06-10

**Authors:** Marijn Rutgers, Daniël BF Saris, Wouter JA Dhert, Laura B Creemers

**Affiliations:** 1 Department of Orthopaedics, University Medical Center Utrecht, Heidelberglaan 100, 3584 CX Utrecht, The Netherlands; 2 Faculty of Veterinary Sciences, Utrecht University, Yalelaan 1, De Uithof, 3584 CL Utrecht, The Netherlands

## Abstract

**Introduction:**

Intraarticular administration of autologous conditioned serum (ACS) recently demonstrated some clinical effectiveness in treatment of osteoarthritis (OA). The current study aims to evaluate the *in vitro *effects of ACS on cartilage proteoglycan (PG) metabolism, its composition and the effects on synovial fluid (SF) cytokine levels following intraarticular ACS administration.

**Methods:**

The effect of conditioned serum on PG metabolism of cultured OA cartilage explants was compared to unconditioned serum. The effect of serum conditioning on levels of interleukin-1beta (IL-1β), IL-4, IL-6, IL-10, IL-13, interferon gamma (IFN-γ), tumor necrosis factor alpha (TNF-α), osteoprotegerin (OPG), oncostatin M (OSM), interleukin-1 receptor (IL-1ra) and transforming growth factor beta (TGF-β) were measured by multiplex ELISA. As TNF-α levels were found to be increased in conditioned serum, the effect of TNF-α inhibition by etanercept on PG metabolism was studied in cartilage explants cultured in the presence of conditioned serum. Furthermore, cytokine levels in SF were measured three days after intraarticular ACS injection in OA patients to verify their retention time in the joint space.

**Results:**

PG metabolism was not different in the presence of conditioned serum compared to unconditioned serum. Levels of the anti-inflammatory cytokines IL-1ra, TGF-β, IL-10 as well as of pro-inflammatory cytokines IL-1β, IL-6, TNF-α and OSM were increased. IL-4, IL-13 and IFN-γ levels remained similar, while OPG levels decreased. TNF-α inhibition did not influence PG metabolism in cartilage explant culture in the presence of condtioned serum. Although OPG levels were higher and TGF-β levels were clearly lower in ACS than in SF, intraarticular ACS injection in OA patients did not result in significant changes in these cytokine levels.

**Conclusions:**

ACS for treatment of osteoarthritis contains increased levels of anti-inflammatory as well as pro-inflammatory cytokines, in particular TNF-α, but conditioned serum does not seem to have a net direct effect on cartilage metabolism, even upon inhibition of TNF-α. The fast intraarticular clearance of cytokines in the injected ACS may explain the limited effects found previously *in vivo*.

## Introduction

Osteoarthritis (OA)-associated cartilage degradation is mediated in part by cytokines and growth factors, excreted into the intraarticular environment by synoviocytes, activated immune cells, or by the articular cartilage itself [1,2]. Therapies interfering with these cytokines may influence disease progression and improve the patient's quality of life.

A pivotal role in the progression of OA has been assigned to the pro-inflammatory cytokine interleukin-1β (IL-1β), which induces a cascade of inflammatory and catabolic events including the expression of cartilage degrading matrix metalloproteinases (MMP) [3], nitric oxygen (NO) production and prostaglandin E_2 _(PGE_2_) release [4], while inhibiting proteoglycan and collagen synthesis [5,6]. The number of type-1 IL-1 receptors is significantly increased in OA chondrocytes [7] and synovial fibroblasts [8], increasing the susceptibility for IL-1α and IL-1β mediated effects. In addition, it was suggested that in OA synovium, a relative deficit in IL-1ra-production exists [1]. As intraarticular administration of recombinant human interleukin-1 receptor antagonist has been shown to alleviate symptoms in several animal models of OA and rheumatoid arthritis [9-11], intraarticular treatment with IL-1ra was also suggested as a feasible treatment for patients with OA.

One example of a disease-modifying osteoarthritis-drug (DMOAD) based on blocking the intraarticular effects of IL-1 associated with OA, is autologous conditioned serum (ACS or Orthokine^®^; Orthogen, Düsseldorf, Germany). Autologous conditioned serum (ACS) treatment consists of six repetitive injections of ACS over a period of 21 days. ACS is prepared from whole blood that is incubated in the presence of glass beads to initiate monocyte activation [12,13]. The resulting *conditioned serum *(ACS), has been shown to contain increased levels of IL-1ra as well as IL-4 and IL-10 [12]. In horses with arthroscopically induced osteochondral defects, ACS treatment demonstrated a reduction in lameness and a decrease in synovial membrane hyperplasia [14]. ACS treatment of human OA patients, however, demonstrated limited to moderate clinical effects [15,16]. Despite the fact that this approach has already been introduced in the clinic, the mechanisms by which administration of this product may result in reduction of OA symptoms is not yet fully understood [14,16,17]. Although the primary goal of ACS treatment is alleviation of OA symptoms, one of the mechanisms may be enhancement of cartilage integrity through the inhibition of inflammatory activity, in particular with respect to Il-1 signalling. In fact, the direct effect of the entire blend of known and unknown factors present in ACS on cartilage metabolism in human OA cartilage has not been described. Moreover, only limited data are available on the actual composition of the conditioned serum. Besides IL-1ra, growth factors, such as transforming growth factor beta 1 (TGF-β1), which stimulates chondrocyte proliferation [18,19], are upregulated during incubation [17]. Of several pro-inflammatory cytokines like IL-1β, tumour necrosis factor-alpha (TNF-α) [20,21] and IL-6 [22], of the last of which also anti-inflammatory effects have been described [23], it is not entirely clear if their levels remain equal or are upregulated during incubation [12,17]. As a consequence of monocyte activation during incubation of blood, anti-inflammatory cytokines such as IL-13, which was shown to inhibit the production of IL-1β and enhance production of IL-1ra [24], and osteoprotegerin (OPG) [25], which protected cartilage in a murine model of surgically induced osteoarthritis from further degeneration [26], may be upregulated. Also pro-inflammatory cytokines oncostatin-M (OSM) [27] and interferon-gamma (IFN-γ) [28] which act synergistically with IL-1β to stimulate production of MMPs and aggrecanases, may be upregulated together with the anti-inflammatory cytokines. Even if the composition of ACS would be favourable to cartilage regeneration, it is still unknown to what extent the intraarticularly injected cytokines are present long enough in the knee joint to exert their actions. The intraarticular availability of adequate levels of IL-1ra is important, as IL-1β is considered to be active at low concentrations and relatively high levels of IL-1ra are required to inhibit the effects of IL-1β [29]. *In vivo*, increased IL-1ra levels were found in equine synovial fluid 35 days after the last (of four) injections with ACS [14].

The current study aims to evaluate the direct *in vitro *effect of conditioned serum on cartilage proteoglycan metabolism, to further evaluate the composition of ACS and to examine to what extent intraarticular injection of ACS is reflected in cytokine level changes in human osteoarthritic synovial fluid.

## Materials and methods

### Preparation of conditioned serum

To prepare conditioned serum, 35 ml of whole blood was acquired through venapunction and aspirated in six polypropylene syringes (5 ml) containing glass beads (Orthogen, Düsseldorf, Germany). The syringes were incubated at 37°C for six hours. After incubation, the blood was centrifuged at 1,000 × g for 10 minutes, and serum was aspirated and stored at -80°C until further use. Control syringes containing whole blood (5 ml without glass beads) were centrifuged and serum was stored at -80°C.

### Effect of conditioned serum on proteoglycan metabolism

To measure the effects of conditioned serum on proteoglycan metabolism, 48 full thickness osteoarthritic cartilage explants were taken of the femoral condyles of OA patients undergoing a total knee arthroplasty (Kellgren-Lawrence grade III) and satisfying the OA criteria of the American College of Rheumatology [30]. The explants were cultured in the presence of conditioned serum (n = 24) or non-conditioned control serum (n = 24) of healthy serum donors. The cartilage was washed, cut into cubes of approximately 3 × 3 × 3 mm, weighed and cultured for 16 days in Dulbecco's Modified Eagles Medium containing 1% penicillin/streptomycin, 1% ascorbic acid (ASAP) and either 25% conditioned serum or 25% control (non-stimulated) serum. The experiment was repeated with two other OA cartilage and serum donor combinations (Table 1).

**Table 1:** Patient characteristics of cartilage explant experiments

Experiment series	Cartilage donor	OA grade (Kellgren-Lawrence)	Cartilage explants/condition	Serum donor
control vs CS	56 year old male	III	24 (48)	26 year old male
	65 year old female	IV	24 (48)	33 year old male
	76 year old male	III	24 (48)	30 year old male

control vs CS vs etanercept	54 year old male	III	8 (24)	27 year old male
	55 year old male	III	8 (24)	27 year old male
	44 year old male	IV	8 (24)	26 year old female

CS, conditioned serum; OA, osteoarthritis.

### Effect of TNF-α inhibition by etanercept on proteoglycan metabolism in the presence of conditioned serum

In another series of three experiments comparing the effect of conditioned serum and unconditioned serum on *in vitro *cartilage metabolism, Etanercept (Enbrel^®^, Wyeth Pharmaceuticals Inc., Collegeville, PA, USA) was added to full-thickness cartilage explants of femoral condyles of OA patients undergoing a knee replacement surgery and cultured *in vitro*. The explants were cultured with 25% control serum (n = 8), 25% conditioned serum (n = 8) and 25% conditioned serum with etanercept (1 μg/ml etanercept, n = 8). This concentration was based on a previous publication showing that this concentration was capable of inhibiting the activity of 40 ng/ml of TNFα [31].

^35^S incorporation was measured by means of a four-hour incubation with ^35^SO_4_^2-^, on Day 4 for all conditions (see below). The medium released on Days 4, 8, 12 and on Day 16 was analysed for proteoglycan release, including release of newly synthesised PGs. The experiment was repeated with two other OA cartilage and serum donor combinations (Table 1).

### ^35^S incorporation

At Days 4, 8 and 12 of the culture, ^35^SO_4_^2-^incorporation (Na_2 _^35^SO_4_, carrier-free; Perkin Elmer, Boston, MA, USA) was measured in order to quantify proteoglycan incorporation by means of a four-hour incubation in culture medium containing 20 μCi of ^35^SO_4_^2-^. For the control as well as for the conditioned serum cultured cartilage explants, eight separate cartilage explants were used for each incorporation time point. Explants were then rinsed in plain culture medium during three 45-minute changes, and the culture of these explants was continued in isotope-free medium until the end of culture on Day 16. At Days 4, 8, 12 and 16, conditioned media were collected to quantify the release of newly synthesised PG. ^35^SO_4_^2- ^incorporation was quantified using a scintillation counter (Tri-carb 1900CA, Packard, Ramsey, MN, USA), and results were normalized to DNA content and weight of the sample.

### Alcian blue immunoprecipitation and DNA assay

On Day 16, all cartilage explants were washed three times in phosphate-buffered saline (PBS) at 4°C. Explants were then digested in 2% papain (Sigma, St. Louis, MO, USA) in 50 mM phosphate buffer, 2 mM N-acetylcysteine, and 2 mM Na_2_-EDTA (pH 6.5) at 65°C for two hours. Part of the digest was used to measure DNA content and part was used for the quantification of the glycosaminoglycan content as a measure of proteoglycan content using an Alcian Blue precipitation assay (described below). Another part was used to measure ^35^SO_4_^2- ^activity.

Glycosaminoglycans (GAGs) were precipitated from the explant digests as well as from the culture medium and stained with an Alcian blue dye solution (Alcian blue 8GX, Sigma-Aldrich, Zwijndrecht, The Netherlands), saturated in 0.1 M sodium acetate buffer, containing 0.3 M MgCl_2 _(pH 6.2) for 30 minutes at 37°C [32]. The blue staining of the medium was quantified photospectrometrically from the change in absorbance at 620 nm, using chondroitin sulphate (Sigma) as a reference. DNA was stained with the fluorescent dye Hoechst 33258 (Sigma) and fluorescence was measured on the Cytofluor (MTX Lab Systems, Vienna, VA, USA) [33], using calf thymus DNA (Sigma) as a reference.

### Composition of autologous conditioned serum (ACS)

Whole blood was obtained from 22 OA patients meeting the American College of Rheumatology criteria for OA (mean age 52 years, range 35 to 72). ACS for intraarticular treatment was prepared by whole blood incubation in the presence of ACS-specific glass beads. Unconditioned serum was taken as control.

### Multiplex ELISA

Multiplex ELISA was used for measurement of cytokine levels in conditioned and unconditioned serum and in SF. Earlier validation studies showed high correlation of multiplex ELISA readings with conventional ELISA [34] and demonstrated that multiplex ELISA is suitable for SF analysis [35]. The cytokines measured were IL-1β, IL-4, IL-6, IL-10, IL-13, IFN-γ, OSM and OPG. Measurements and data analysis were performed using the Bio-Plex system in combination with the Bio-Plex Manager software version 3.0 using five parametric curve fitting (Bio-Rad Laboratories, Hercules, CA, USA). Coating antibodies for IL-1β, IL-6, IL-10 and TNF-α were provided by Strathman Biotec (Hannover, Germany); coating antibodies for IL-4, OPG and OSM by R&D Systems (Abingdon, UK), coating antibody for IL-13 by National Institute for Biological Standards and control (Potters Bar, UK) and coating antibody for IFN-γ by BD Biosciences (San Diego, CA, USA). The recombinant proteins for IL-1β, IL-6, IL-10 and IL-13 were provided by Sanquin (Amsterdam, The Netherlands), for IL-4 and IFN-γ by eBioscience (San Diego, CA, USA), for TNF-α by BD Biosciences, for OPG by R&D Systems (Abingdon, UK) and for OSM by Biocarta (Hamburg, Germany).

Preparation of recombinant cytokine mixes, covalent coupling of the captured antibodies to the microspheres and preparation of detection antibodies were performed as described previously [34,35]. For determination of cytokine profiles in SF, aliquots of 200 μl were first pre-treated with 20 μl of hyaluronidase (0.5 mg/ml, type IV-S, Sigma-Aldrich, Zwijndrecht, The Netherlands) for 30 minutes at 37°C, spun over 0.22 μm nylon membrane (Spin-X column; Corning, The Netherlands) and diluted with High-Performance ELISA-buffer (Sanquin Blood Supply Foundation, Utrecht, Netherlands) at a 1:2 dilution. Recombinant protein standards and calibration curves were prepared in serum diluents (R&D Systems). A mix containing 1,000 coupled microspheres per cytokine (total volume of 10 μl/well) was added to the standard, sample or blank, and incubated for 60 minutes. Next, a 10 μl mix of biotinylated antibodies (final concentration 16.6 μg/ml for each antibody) was added to each well and incubated for an additional 60 minutes. Beads were washed in PBS containing 1% BSA and 0.5% Tween 20 (pH 7.4) in order to remove residual sample and unbound antibodies. After incubation for 10 minutes with 0.5 μg/ml streptavidin R-phycoerythrin (BD Biosciences) and washing twice with 1% BSA and 0.5% Tween 20 (pH 7.4), the fluorescence intensity of the beads was measured in 100 μl High Performance ELISA buffer (Sanquin). Measurements and data analysis were performed using the Bio-Plex system in combination with the Bio-Plex Manager software version 3.0 using five parametric curve fitting (Bio-Rad Laboratories). To eliminate the possibility of inter-assay variability, control and conditioned serum samples were measured in duplo in the same assay.

### ELISA

IL-1ra and TGF-β1 levels were measured using commercially available ELISA kits (Quantikine^®^, DRA00 and DB100B, R&D Systems), following the manufacturer's protocol.

### Analysis of cytokines in synovial fluid after ACS injection

Twenty-two OA patients were treated with six consecutive injections of ACS at Days 0, 3, 7, 10, 14 and 21, according to the ACS treatment schedule. To this end, a 21 gauge needle was inserted into the knee joint through a lateral supra-patellar approach. After aspiration of the SF, 2 ml of ACS was injected into the joint through a 0.22 mm sterile nitrocellulose filter (Millex^®^, Millipore Express, Carrigtwohill, Co. Cork, Ireland). The knee was flexed and extended manually to ensure thorough distribution of the serum throughout the joint. Within 30 minutes after aspiration, the aspirated SF was centrifuged for 10 minutes at 1,000 × g and the aspirate and the residual serum samples were stored at -80°C until further analysis.

Treatment of patients with ACS was performed in compliance with the Helsinki Declaration. Written informed consent was given by all participants, and approval by the Medical Ethics Committee (University Medical Center Utrecht, The Netherlands; trial registration ID 03-232/G-O) was obtained before initiation of the trial.

### Statistical analysis

SPSS version 15.0 for Windows (SPSS Inc., Chicago, IL, USA) was used for data analysis. Paired *t*-tests were used to compare cytokine levels in ACS and control serum of OA patients (n = 22 patients), and independent *t*-tests were used to compare PG content, DNA content and PG/DNA for each of the *in vitro *experiments. Analysis of variance (ANOVA) was performed on the pooled data of the experiments, with randomised block design to correct for inter-donor variability. Repeated measurement analysis was used to identify changes in SF cytokine levels during treatment. Comparisons between different treatments (control, ACS, Etanercept) were followed by a Bonferroni correction. *P*-values less than 0.05 were considered statistically significant. Graphs show mean values with standard deviation (SD).

## Results

### Proteoglycan metabolism of cartilage explant culture

**Figure 1:**
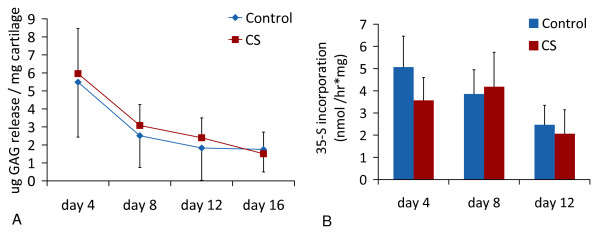
**Proteoglycan incorporation and release during culture of cartilage explants (mean +/- SD)**. **(a) **Proteoglycan release during culture of cartilage explants. A similar amount of proteoglycans were released into the culture medium by explants cultured with unstimulated (n = 24) or conditioned serum (CS; n = 24). **(b) **PG incorporation rate on Days 4, 8 and 12 of the culture, measured using ^35^SO_4_^2- ^incorporation (n = 8 per timepoint). Results are representative of three separate experiments with different OA cartilage donor - serum donor combinations.

**Figure 2:**
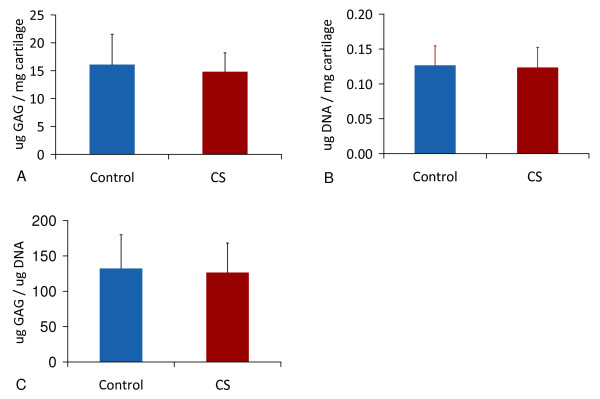
**Proteoglycan and DNA content during culture of cartilage explants (mean +/- SD)**. Proteoglycan and DNA content of cartilage explants after culturing with unconditioned serum (n = 24) or conditioned serum (CS, n = 24 cartilage explants). **(a) **Proteoglycan content. **(b) **DNA content, **(c) **PG/DNA ratio.

An average of 40% of explant proteoglycan (PG) was released into the culture medium in 25% conditioned serum or in 25% control serum (Figure 1). PG release, PG content at the end of the culture, nor ^35^S incorporation on Days 4, 8 or 12 differed between OA cartilage explants cultured in either condition, measured with independent *t*-tests and ANOVA (Figures 1 and 2).

### Proteoglycan metabolism upon TNF-α inhibition by etanercept

**Figure 3:**
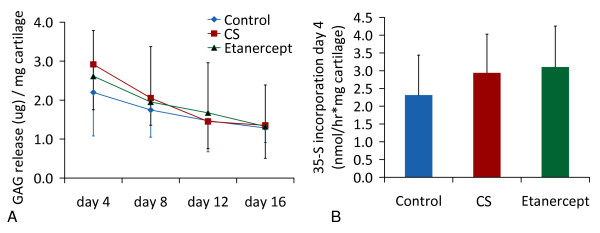
**Proteoglycan incorporation and release during culture in unconditioned, conditioned or conditioned serum with Etanercept (mean +/- SD)**. Proteoglycan release and incorporation (mean +/- SD) in the presence of unconditioned serum (control, n = 8), conditioned serum (CS, n = 8) or conditioned serum with etanercept (n = 8). **(a) **Proteoglycan release, **(b) **Proteogycan incorporation on Day 4, measured by ^35^SO_4_^2- ^incorporation (n = 8). Results are representative for three separate experiments with different OA cartilage donor - serum donor combinations.

**Figure 4:**
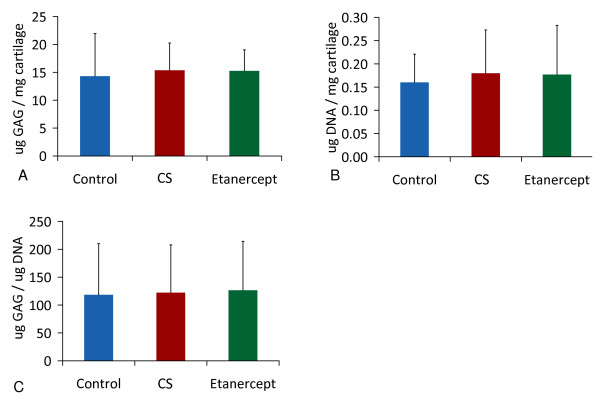
**Proteoglycan and DNA content during culture in unconditioned, conditioned serum or conditioned serum with Etanercept (mean +/- SD)**. Proteoglycan (PG) metabolism in the presence of unconditioned serum (control, n = 8), conditioned serum (CS, n = 8) or conditioned serum with etanercept (n = 8). **(a) **PG content, **(b) **DNA content, **(c) **PG/DNA ratio.

Addition of etanercept to conditioned serum or control serum did not alter PG release, PG incorporation and final PG or DNA content after culture measured with independent *t*-tests and ANOVA (Figures 3 and 4).

### Cytokines in unconditioned serum and ACS

**Figure 5:**
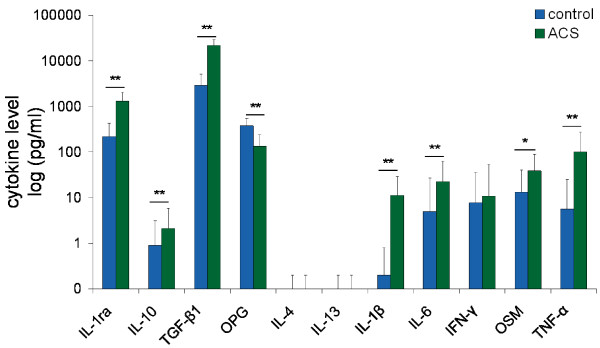
**Effects of whole blood conditioning on serum cytokine levels of 22 OA patients**. Cytokine levels in serum of 22 OA patients, before incubation (control) and after six hours of incubation in the presence of glass beads (ACS). Note the increase in anti-inflammatory cytokines (IL-1ra, TGF-β1, IL-10) and pro-inflammatory cytokines (IL-1β, IL-6, IFN-γ, OSM, TNF-α) after incubation. OPG levels were decreased. All values are displayed as mean ± SD in pg/ml. * *P *< 0.01; ** *P *< 0.001.

Serum levels of IL-10 and IL-1ra increased after conditioning (3.0-fold and 7.9-fold, respectively) (*P *< 0.01). Of the other anti-inflammatory cytokines, TGF-β1 was upregulated (14.9-fold) and OPG was downregulated (2.8-fold) (both *P *< 0.001). Pro-inflammatory cytokines IL-1β, OSM and TNF-α were upregulated (20.9-fold, 2.9 fold and 10.2-fold, respectively) (all *P *< 0.01) while IFN-γ levels did not change. IL-6 levels were upregulated 19.3 fold (*P *< 0.001). IL-4 levels and IL-13 levels were below detection limits in non-stimulated serum as well as in ACS (Figure 5).

### Cytokines in synovial fluid before and after treatment

Sufficient amounts of SF for all treatment time points were available for analysis in 14 patients. To verify whether this implied a bias in the ensuing experiments, clinical grade of OA and baseline serum cytokine levels were compared between this group of patients and the eight patients from whom no SF could be aspirated. No statistically significant differences between these patients and the other group of eight patients were noted (Table 2).

**Table 2:** Clinical scores and serum cytokine levels in patients with sufficient and with non-sufficient SF for analysis

	KOOS score	KSCRS score	Baseline serum cytokine levels									
			
			IL-1	IL-4	IL-6	IL-10	IL-13	TNFα	IFNγ	OSM	OPG	IL-1ra
SF available and analysed (14 patients)	46 (10)	79 (19)	0.2 (0.7)	0.0 (0.0)	8.5 (28)	1.5 (2.7)	0.0 (0.1)	0.0 (0.2)	2.2 (2.2)	13 (27)	376 (128)	180 (137)

No SF available (8 patients)	51 (15)	75 (15)	0.2 (0.6)	0.0 (0.0)	0.1 (0.2)	0.1 (0.2)	0.1 (0.2)	14 (29)	16 (44)	14 (29)	371 (229)	276 (288)

Pre-treatment clinical OA and baseline serum characteristics (mean +/- SD) of patients whose synovial fluid were analysed, were similar to those of patients whose synovial fluid were not analysed due to insufficient amounts of SF in the knee at time of aspiration.

KOOS, Knee and Osteoarthritis Outcome Score; KSCRS, Knee Society Clinical Rating Scale; SF, synovial fluid; OA, osteoarthritis; IL-4, interleukin-4; IL-6, interleukin-6; IL-10, interleukin-10; IL-13, interleukin-13; IFN-γ, interferon gamma; OSM, oncostatin M; TNF-α, tumor necrosis factor alpha; OPG, osteoprotegerin; IL-1ra, interleukin-1 receptor antagonist.

**Figure 6:**
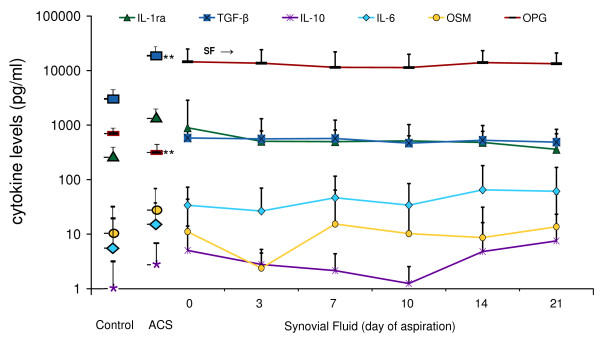
**Cytokine levels in control serum and ACS, and in synovial fluid during treatment**. Control serum (control), autologous conditioned serum (ACS) and synovial fluid cytokine levels (mean +/- SD) of IL-1RA, TGF-β1, IL-10, IL-6, OSM and OPG during treatment with ACS in 14 OA patients. The large symbols next to the y-axis correspond to the levels of these cytokines in control serum and the injected ACS. TGF-β1 levels in SF were lower than in the injected ACS, and OPG levels in SF were higher than in ACS (*P *< 0.01). During the course of treatment, no significant changes in cytokine levels occurred despite repeated ACS injection (SF was aspirated before each of the six injections with ACS, at t = 0, Day 3, Day 7, Day 10, Day 14 and Day 21).

Levels of IL-1β, IL-4, IL-13, TNF-α, and IFN-γ were low or undetectable in SF before and during treatment with ACS. IL-6, OSM, OPG, IL-10, TGF-β and IL-1ra were detectable in synovial fluid, but only OPG and TGF-β levels differed significantly from ACS levels. The levels of OPG in SF at baseline were higher than in ACS (14,476 pg/ml vs. 134 pg/ml, *P *< 0.001), but had not changed significantly three days after injection of the serum. Baseline synovial fluid TGF-β levels were lower than in ACS (580.7 vs. 21,670.9 pg/ml, *P *< 0.001), but did not change significantly after ACS injection either (Figure 6).

## Discussion

Disease-modifying drugs for conservative treatment of osteoarthritis have proven effective in a variety of randomized controlled clinical trials [36,37]. Although autologous conditioned serum (ACS, Orthokine^®^) proved slightly to moderately effective for alleviation of OA symptoms up to two years after treatment in human OA patients [15,16], many aspects of this therapy have remained unclear so far.

*In vitro*, conditioned serum does not seem to have a direct effect on cartilage metabolism compared to unstimulated serum. In line with earlier studies, IL-1ra levels of ACS in the current study were upregulated, although the reported relative increases in conditoned serum differed an order of a magnitude with those from the current study [12,17]. Also IL-10 levels were upregulated two-fold as found earlier [4], but IL-4 was hardly detectable. It is not known to what extent this is related to the change in the manufacturer's protocol, in which the conditioning period is reduced to six hours [17], as opposed to the 24 hours initially included in the preparation protocol [14,15]. Allegedly, most of the cytokine production occurs after six hours. Moreover, although this has not been argued as such by the manufacturer, long incubation periods at body temperature are known to reduce the bioactivity of most cytokines, while their immunoreactivity as determined by ELISA is still retained [38]. In particular Il-10, one of the anti-inflammatory cytokines upregulated in ACS, has been shown to have a half-life of several hours under these conditions [39]. This may also represent another explanation for the limited effects found *in vivo *thus far. More important, however, pro-inflammatory cytokines, in particular IL-1β and TNF-α, were found to be significantly upregulated in the current ACS study, in contrast to previous results [17]. As, unlike for IL-1, the increased TNF-α levels were not counterbalanced by an increase in levels of natural inhibitors, and TNF-α has been postulated to have degenerative effects on cartilage [21,40], this may have explained the limited effects found in OA patients treated with conditioned serum. However, blocking the action of TNF-α [31] did not result in a net positive effect of conditioned serum on matrix metabolism *in vitro*, suggesting that, if any, *in vivo *effects of the TNFα in the injected ACS would have been indirect. It is not clear to what extent the increased levels of IL-6, OSM and lower levels of OPG in conditioned serum may have had a pro-inflammatory effect, but as conditioned serum addition did not result in decreased sulphate incorporation after four days of cartilage explant culture, or a lower PG content after 16 days of culture, conditioning of serum is not likely to have any effect on OA cartilage. These findings were strengthened by the large number of explants used per experiment, and by repeating both experiments in a total of six different OA donors. Nevertheless, it cannot be excluded that factors present in conditioned serum, either known or as yet undiscovered, play a role *in vivo *by inducing other mediators, not determined in the current study, in the joint space. With respect to the role of Il-1 signalling in OA, in the one human clinical study using recombinant IL-1ra as a treatment for OA, a single injection into the knee joint did not result in an improvement of OA symptoms [41]. This may have been due to fast clearance from the joint space. Injection of ACS led to an increase of IL-1ra SF levels in osteoarthritic equine knee joints during ACS treatment *in vivo *[14]. However, in the current study, IL-1ra levels did not increase during the course of the treatment, even though the interval between injection and measurement was shorter than in the former study (3 days vs 7 and 35 days [14]). The fast clearance of injected cytokines from the joint found in the current study suggests that any *in vitro *net effect would still have been difficult to reproduce *in vivo*. Continuous intraarticular availability of IL-1ra may be more effective. *In vivo *injection of synoviocytes transduced with the IL-1ra gene into a canine knee joint after sectioning of the anterior cruciate ligament [11] and intraarticular injection of IL-1ra plasmid into a rabbit knee joint after meniscectomy resulted in reduction of OA clinical symptoms (histological parameters, preservation of articular cartilage quality) [42]. Eventually these long term approaches may be more effective than the limited number of injections of this treatment, but currently they are not practically feasible in a clinical setting. Nevertheless, even if IL-1ra levels are increased in the synovial fluid *in vivo*, it is uncertain if these IL-1ra levels correlate with OA symptoms or disease progression, as the ratio of IL-1ra to IL-1β in the SF of human OA subjects were shown not to correlate with pain or with the Lequesne OA index [43].

With respect to the upregulation of IL-1β, its role in progression of OA may actually be disputed [44]. In our study, IL-1β levels in OA SF were extremely low, which is in line with previous reports [34,45,46]. Although there are studies in which IL-1β inhibited proteoglycan synthesis *in vitro *at concentrations as low as 10 pg/ml [47], commonly IL-1β concentrations of at least 1,000 pg/ml are used to induce detectable cartilage damage [48,49]. Studies demonstrating synergistic effects of IL-1β with IFN-γ, TNF-α, IL-17 or Oncostatin-M [50] also departed from IL-1β concentrations much higher than detected in OA synovial fluid and hence synergistic effects of low IL-1β levels with other cytokines in the current study do not seem likely. Moreover, the baseline IL-1ra levels in the SF of the currently studied OA patients were already in the effective range to block IL-1β [29]. Although it may be argued that the patients with sufficient amounts of SF may have differed from the group as a whole, this is contradicted by the observation that serum cytokine levels as well as the clinical response of the patients with sufficient amounts of SF were similar to the patients with insufficient amounts of SF.

A high standard deviation in synovial fluid cytokine levels was encountered, as is common in OA. Increasing the number of subjects may decrease this standard deviation and possibly enable subgroup analysis (for example, by progression of OA according to radiological parameters (dGEMRIC) or by clinical parameters (KOOS scores). However, as the group of patients was already small, this would have even further reduced the likelihood of finding statistically significant changes.

Future evaluation of intraarticular cytokine changes following ACS injection might include SF analysis earlier after injection, which would give further insight on intraarticular half-life. Also, effects of ACS on synovium, either alone or in coculture with cartilage explants may be studied. Even though multiplex ELISA showed good to excellent correlation with ELISA [34], separate ELISAs may give slightly more accurate information about absolute cytokine levels.

Although the present *in vitro *data show no effect of ACS and a short intraarticular half life, a recent clinical study demonstrated a two-year lasting improvement of ACS treatment compared to hyaluronic acid and placebo treatment [51]. However, it must be noted that the treatment regimens differed, with six injections of ACS being compared to three injections of hyaluronic acid or placebo. Moreover, as none of the clinical trials carried out so far included unconditioned serum as a placebo, it can actually not be excluded that injection of serum without prior conditioning per se has a beneficial effect on proteoglycan metabolism *in vivo*.

## Conclusions

In conclusion, ACS is a mix of counteracting growth factors and cytokines that does not have a direct effect on cartilage metabolism and probably has a minimal influence in the joint space, given the fast disappearance of cytokines from the synovial fluid after injection. Development of new intraarticular therapies may focus on their prolonged presence in the joint space.

## Abbreviations

ACS: autologous conditioned serum; ASAP: ascorbic acid; DMOAD: disease-modifying osteoarthritis-drug; GAGS: glycosaminoglycans; IFN-γ: interferon gamma; IL-1ra: interleukin-1 receptor antagonist; IL-1β: interleukin-1 beta; IL-4: interleukin-4; IL-6: interleukin-6; IL-10: interleukin-10; IL-13: interleukin-13; MMP: matrix metalloproteinases; NO: nitric oxygen; OA: osteoarthritis; OPG: osteoprotegerin; OSM: oncostatin M; PG: proteoglycan; PGE_2_: prostaglandin E_2_; SD: standard deviation; SF: synovial fluid; TNF-α: tumor necrosis factor alpha; TGF-β: transforming growth factor beta.

## Competing interests

The authors declare that they have no competing interests.

## Authors' contributions

MR carried out the *in vitro *and *in vivo *experiments, analysed and interpreted the data and drafted the manuscript. DS participated in design and coordination of the study and revised the manuscript. WD was involved in design of the study and revised the manuscript. LC conceived of the study and participated in its design and coordination, aided with statistics and revised the manuscript. All authors read and approved the final manuscript.
